# The effects of two weeks high-intensity interval training on fasting glucose, glucose tolerance and insulin resistance in adolescent boys: a pilot study

**DOI:** 10.1186/s13102-019-0141-9

**Published:** 2019-12-09

**Authors:** Emma J. Cockcroft, Bert Bond, Craig A. Williams, Sam Harris, Sarah R. Jackman, Neil Armstrong, Alan R. Barker

**Affiliations:** 1 Children’s Health and Exercise Research Centre, Sport and Health Sciences, College of Life and Environmental Sciences, University of Exeter, Exeter, EX1 2LU UK; 2 College of Medicine and Health, University of Exeter, Exeter, EX1 2LU UK; 3 Sport and Health Sciences, College of Life and Environmental Sciences, University of Exeter, Exeter, EX1 2LU UK

**Keywords:** Cardiometabolic health, Aerobic fitness, Physical activity, Youth

## Abstract

**Background:**

Current evidence of metabolic health benefits of high-intensity interval training (HIIT) are limited to longer training periods or conducted in overweight youth. This study assessed 1) fasting and postprandial insulin and glucose before and after 2 weeks of HIIT in healthy adolescent boys, and 2) the relationship between pre intervention health outcomes and the effects of the HIIT intervention.

**Methods:**

Seven healthy boys (age:14.3 ± 0.3 y, BMI: 21.6 ± 2.6, 3 participants classified as overweight) completed 6 sessions of HIIT over 2 weeks. Insulin resistance (IR) and blood glucose and insulin responses to a Mixed Meal Tolerance Test (MMTT) were assessed before (PRE), 20 h and 70 h after (POST) the final HIIT session.

**Results:**

Two weeks of HIIT had no effect on fasting plasma glucose, insulin or IR at 20 h and 70 h POST HIIT, nor insulin and glucose response to MMTT (all *P* > 0.05). There was a strong negative correlation between PRE training IR and change in IR after HIIT (*r* = − 0.96, *P* < 0.05).

**Conclusion:**

Two weeks of HIIT did not elicit improvements to fasting or postprandial glucose or insulin health outcomes in a group of adolescent boys. However the negative correlation between PRE IR and improvements after HIIT suggest that interventions of this type may be effective in adolescents with raised baseline IR.

## Background

Insulin resistance (IR), impaired beta cell function (%β) and glucose tolerance are all implicated in the development of type two diabetes (T2D) and cardiovascular disease (CVD) [1]. Such risk factors are known to be prevalent in youth [2] and can predict future risk of CVD and T2D [3]. The early development of IR begins 10–20 y before onset of T2D and is thought to be one of the best predictors of future diabetic risk [4]. This makes the pubertal years a prime target for interventions to prevent the onset of T2D and CVD, as well as associated co-morbidities.

Physical activity (PA) is an effective intervention to improve risk factors associated with T2D and CVD in youth. Meta-analysis has shown a small to moderate effect of exercise training to improve fasting insulin and IR in youth, especially for those who are overweight or obese [5], with aerobic exercise training associated with reductions in fasting insulin and HOMA-IR [6]. However, despite the known importance of PA in youth, less than one third of school aged children and adolescents meet the minimum UK government recommendation of 60 min of moderate to vigorous physical activity (MVPA) per day [7]. Furthermore, a meta-analysis of school-based interventions designed to increase levels of PA in adolescents showed a small but non-significant increase in moderate-to-vigorous physical activity equating to approximately two additional minutes of MVPA per day [8]. Adolescence is also associated with declining levels of PA [9] and represents a period in time when PA has the most profound effect on IR [10], highlighting the importance of exploring alternative “time efficient” forms of PA to improve cardiometabolic health outcomes in this group.

Recent observational data in youth have shown that small amounts (< 7 min) of vigorous intensity PA are associated with favourable temporal changes in cardiometabolic risk factors, including blood pressure, waist circumference and aerobic fitness in youth [11]. This suggests that promoting high-intensity PA in this group may help in modifying disease risk. In healthy adolescents, just 2 weeks high-intensity interval training (HIIT), consisting of 4 to 7 short duration (30 s) sprint intervals, has been shown to improve aerobic fitness [12], indicating that short duration HIIT may have health benefits in youth. However evidence for the metabolic health benefits of HIIT in youth is currently limited to longer (7–12 weeks) training periods that often target adolescents who are overweight or have low aerobic fitness [13–17]. In contrast, it has recently been shown that improvements in insulin sensitivity (IS) and glucose tolerance in adolescent boys are possible after just a single bout of high-intensity interval exercise (HIIE) [18], suggesting that repeated bouts of HIIE performed over just 2 weeks may be a feasible way to improve glucose tolerance and IS in youth.

The increased IS following a single session of HIIE has been shown to persist for ~ 48 h in adults [19, 20], and up to 24 h in adolescents [21], meaning that any improvements in health outcomes beyond this time frame may be considered a chronic adaption to training. Studies with both healthy adult participants and patients with T2D have shown an increase in the expression of skeletal muscle glucose transporters (e.g. GLUT-4) and the activity of mitochondrial enzymes after just 1–2 weeks of HIIT [22, 23], suggesting chronic adaptations are possible in this timeframe. However, a recent study has shown that 2 weeks of HIIT in a mixed-sex group of adolescents had no effect on fasting and postprandial plasma insulin and glucose outcomes when measured 24 and 72 h after the last training session [24]. This finding was surprising given previous work showing an single bout of HIIE improved postprandial insulin and glucose outcomes in adolescent boys both immediately [18, 21] and up to 24 h [21] after exercise. The unchanged insulin and glucose may, in part, be due to the combined analysis of the adolescent boys and girls in previous work [24], given that previous research has indicated different exercise effects by sex [25]. Additionally the use of the HOMA method to estimate IR is known to have poorer measurement reliability [26] compared to other indices such as the quantitative insulin sensitivity check index (QUICKI) [27] and fasting glucose:insulin ratio (FGIR) [28]. Establishing the effects of exercise training in boys specifically is important since boys are at an increased risk of developing IR and impaired fasting glucose compared to their female peers [29].

Using a subset of data reported previously [24], the aim of this paper was to examine changes in glucose and insulin outcomes in adolescents boys after 2 weeks of HIIT, both 1 day after (acute) and 3 days after (chronic) the last training session. Representing adaptions as a result of the final training session (acute) and longer terms adaptions as a result of the longer training period (chronic). Secondly we aimed to explore the relationship between pre intervention insulin resistance (IR), BMI and aerobic fitness, and the effects of the HIIT intervention on changes to IR.

## Methods

### Participants

Nine boys were recruited from year 10 of a local secondary school. This sample size was based on the ability to detect a moderate to large mean difference for glucose and insulin outcomes based on previous work examining the acute effect of HIIT [21, 30, 31]. All participants were deemed able to participate in the study by completing an initial health questionnaire to exclude any metabolic or medical conditions that contradict exercise or are known to effect glucose metabolism. Following an explanation of the study procedures and the associated risks and benefits, parental consent and participant assent were obtained. Ethical approval was granted by the University of Exeter sport and health sciences ethics committee. One boy failed to complete the HIIT due to an unrelated illness, and one boy could not complete the training due to an unrelated injury. This left a sample of seven participants (14.3 ± 0.3 y) for analysis.

### Study design

This study consisted of four laboratory visits, and 6 training sessions in the school setting, which took place over a 3 week period. Visits included an initial familiarisation visit and three experimental visits. Visits 1 and 2 consisted of baseline measures of aerobic fitness and the glucose and insulin response to a mixed meal tolerance test (MMTT) prior to undertaking the HIIT intervention (PRE). Visits 1 and 2 were separated by 3–5 days. Participants then completed 6 supervised HIIT sessions over a 2 week period, after which post training measures were assessed 20 h (visit 3; 20 h POST) and 70 h post-intervention (visit 4; 70 h POST).

#### Visit 1: Familiarisation and baseline fitness assessment

Stature and body mass were measured to the nearest 0.01 m and 0.1 kg, and used to calculate body mass index (BMI). BMI was used to classify participants as normal weight, overweight and obese, using validated age-specific percentile cut points [32]. Pubertal status was determined by self-assessment of the five stages of pubic hair development described by Tanner [33].

Participants were familiarised with the cycle ergometer (Lode Excalibur Sport, Groningen, Netherlands) and completed a combined ramp-incremental and supramaximal test to exhaustion to determine maximal oxygen uptake $\left(\dot{V}{\mathrm{O}}_2\kern.5em \max \right)$ and the gas exchange threshold (GET) [34]. Pulmonary gas exchange and heart rate were measured (Cortex Metalyzer III B, Germany) and $\dot{V}$O_2_ max was accepted as the highest 10 s average $\dot{V}$O_2_ during the ramp or supra-maximal test. Peak power (PP) was taken as the highest power output during the ramp test whilst maintaining a cadence > 60 revolutions^.^min^− 1^. The GET was estimated at the point where the first disproportionate increase in VCO_2_ production compared to $\dot{V}$O_2_ and verified using the ventilatory equivalents for $\dot{V}$O_2_ and $\dot{V}$CO_2_.

#### Visits 2: Baseline metabolic assessment

Participants were driven to the laboratory and arrived at ~ 07:45 following a 12 h overnight fast. After 15 min of seated rest, participants provided a capillary blood sample for plasma glucose and insulin. At ~ 08:30 a MMTT was conducted which consisted of a commercially available fruit smoothie with 50 ml of double cream added, chocolate croissant with chocolate spread and a chocolate muffin (80 g of glucose, 68 g of fat, 7134 kJ). The meal was consumed over a 15 min period, after which capillary blood samples were taken at 30, 60, 120 min for assessment of plasma glucose and insulin. No other food was consumed and water was available ad libitum during visit 2 (PRE). This was recorded and subsequently replicated for the POST measures. Participants remained in the laboratory throughout the visit, completing sedentary activities such as reading, watching DVDs or playing computer games. Participants left the laboratory at ~ 15:00.

#### HIIT intervention

Participants performed a 2 week HIIT programme on a cycle ergometer (Monark 827e, Monark exercise AB, Sweden) with adjustments made to the handle bar and seat height for each participant. Training took place within a local secondary school and consisted of 3 supervised HIIT sessions per week. Sessions were carried out during the school lunch break. Each session started with a 3 min warm up of unloaded pedalling, followed by 8–10 one min intervals at 90% of the PP achieved during the incremental ramp test performed during visit 1. Each interval was interspersed with 75 s of unloaded pedalling. This HIIT protocol was selected to mimic previous studies from our laboratory [18, 35, 36]. Sessions one and two consisted of 8 × 1 min bouts, sessions three and four 9 × 1 min bouts and sessions five and six 10 × 1 min bouts. Participants were asked to maintain a self-selected cadence (70–95 revolutions^.^min^− 1^) and were reminded of this during each session.

#### Visit 3 and 4: post-training

The protocol outlined above for visit 2 was replicated the day after (20-POST) and 3 days (70-POST) after the last training session. One hour after completion of the MMTT during the 70- POST visit, participants completed a post intervention $\dot{V}$O_2_ max assessment as described in visit 1.

### Standardisation of physical activity and diet

Physical activity was measured during the 48 h period prior to each experimental visit using a wrist worn accelerometer (GENEActiv, Activinsights, UK). For visit three this 48 h period included the final training session. Time spent performing, light, moderate and vigorous PA was determined using cut points previously validated in a paediatric population [37]. Participants were asked to avoid any structured physical activity outside of the training intervention and before any laboratory visits.

With supervision from their parents/guardians, a food diary was completed by each participant during the 48 h period preceding each experimental visit. Food diaries were assessed to estimate total energy and macronutrient content using commercially available software (CompEat Pro, Nutrition systems, UK). Participants were asked to replicate their diet during the 48 h preceding each experimental visit and if appropriate, to document any discrepancies.

### Blood analyses

Fingertip capillary blood samples (~ 600 μL) were taken from a pre-warmed hand into a fluoride heparin coated and lithium heparin coated microvette (CB 300 tubes, Sarstedt Ltd., Leicester, UK) for plasma glucose and insulin determination, respectively. Both microvettes were centrifuged at 6000 revolutions.min^− 1^ for 10 min. Plasma was separated for immediate analysis of glucose (YSI 2300 Stat Plus Glucose analyser, Yellow Springs, OH, USA) or stored at –80 °C for later analysis of plasma insulin using an ELISA enzyme immunoassay kit (DRG Diagnostics, Germany). In our laboratory, the within batch coefficients of variation for the plasma insulin and glucose analyses were < 5%.

### Data handling

Changes in plasma glucose and insulin during the postprandial period following the MMTT were quantified using total and incremental area under the curve (tAUC, iAUC) [38] calculated using the trapezium rule (GraphPad Prism, GraphPad, SanDiego, CA). tAUC is related to basal blood glucose and reflects the amplitude of change. iAUC more accurately describes the glycaemic response to MMTT, and the dynamic change over time, independent of baseline value. Fasting plasma glucose and insulin were used to calculate IR, IS and %β using using HOMA-IR [39], QUICKI [27] and FGIR [28], which have been validated for use in adolescents [40].

### Statistical analysis

Descriptive statistics were calculated using SPSS (version 19.0, Chicago, USA) and presented as mean ± SD. Analysis of the HOMA, QUICKI, FGIR, fasting glucose and insulin, and tAUC and iAUC response to the MMTT across visits was performed using a one-way repeated-measures ANOVA, follow up comparisons between time points (PRE, 20 h-POST and 70 h-post) were only carried out if there was a significant main effect in the ANOVA. The Eta squared thresholds of 0.01, 0.06 and 0.14 were used to identify a small, moderate and large effect from the ANOVA analyses. Changes in aerobic fitness parameters were assessed by a paired sample t-test. The magnitude of the difference between variables of interest were explored using ES [41].

To understand the influence of different parameters on the effectiveness of the HIIIT intervention and understand the relationship between pre intervention health outcomes and the effects of HIIT intervention, Pearson’s correlations were performed between HOMA-IR, QUICKI, FGIR, V̇O2 max and BMI at baseline (PRE) and change in HOMA-IR after the 2 week training period (20 h-POST). A significant correlation was accepted if *P* < 0.05.

## Results

The participants’ descriptive characteristics are shown in Table 1. Maturity status as described by pubic hair was provided by 6 participants and ranged between stages 3 and 4 (stage 4: *n = 4*, stage 3: *n = 2*). The BMI of participants ranged from 17.8 to 24.0 kg∙m^− 2^, with 3 participants classified as overweight according to age and gender specific cut points ^26^. Time spent in moderate and vigorous PA in the 48 h preceding each visit highlighted no differences between visits (*P* > 0.05). No differences in estimated energy intake or macronutrient contribution to diet were evident prior to each visit (all *P* > 0.05). The PA and diet data are shown in Table 2.

**Table 1:** Participant descriptive characteristics

	Mean ± SD	Range
Age, y	14.3 ± 0.3	13.9–14.7
Body mass, kg	60.0 ± 7.4	57.7–69.9
Stature, m	1.67 ± 0.81	1.57–1.78
BMI, kg.m^− 2^	21.6 ± 2.6	17.8–24.6

Results expressed as mean ± SD and range

*BMI* body mass index

**Table 2:** Physical activity and dietary intake during the 48 h preceding each experimental visit

	PRE	20 h POST	70 h POST	ANOVA *P*-value
Moderate-vigorous physical activity (min∙day ^− 1^)	45 ± 25	59 ± 42	56 ± 17	0.55
Total energy intake (kcal∙day ^− 1^)	1971 ± 280	1950 ± 294	2052 ± 293	0.71
Energy from carbohydrate (%)	43 ± 7	47 ± 5	47 ± 9	0.67
Energy from fat (%)	40 ± 10	36 ± 4	38 ± 5	0.54
Energy from protein (%)	18 ± 4	17 ± 4	14 ± 4	0.43

Results shown as Mean ± SD. Twenty hour POST includes the final training session of the HIIT intervention (~ 27 min)

All participants completed the six HIIE training sessions, with 100% adherence to the protocol, with no adverse effects recorded.

**Fig. 1:**
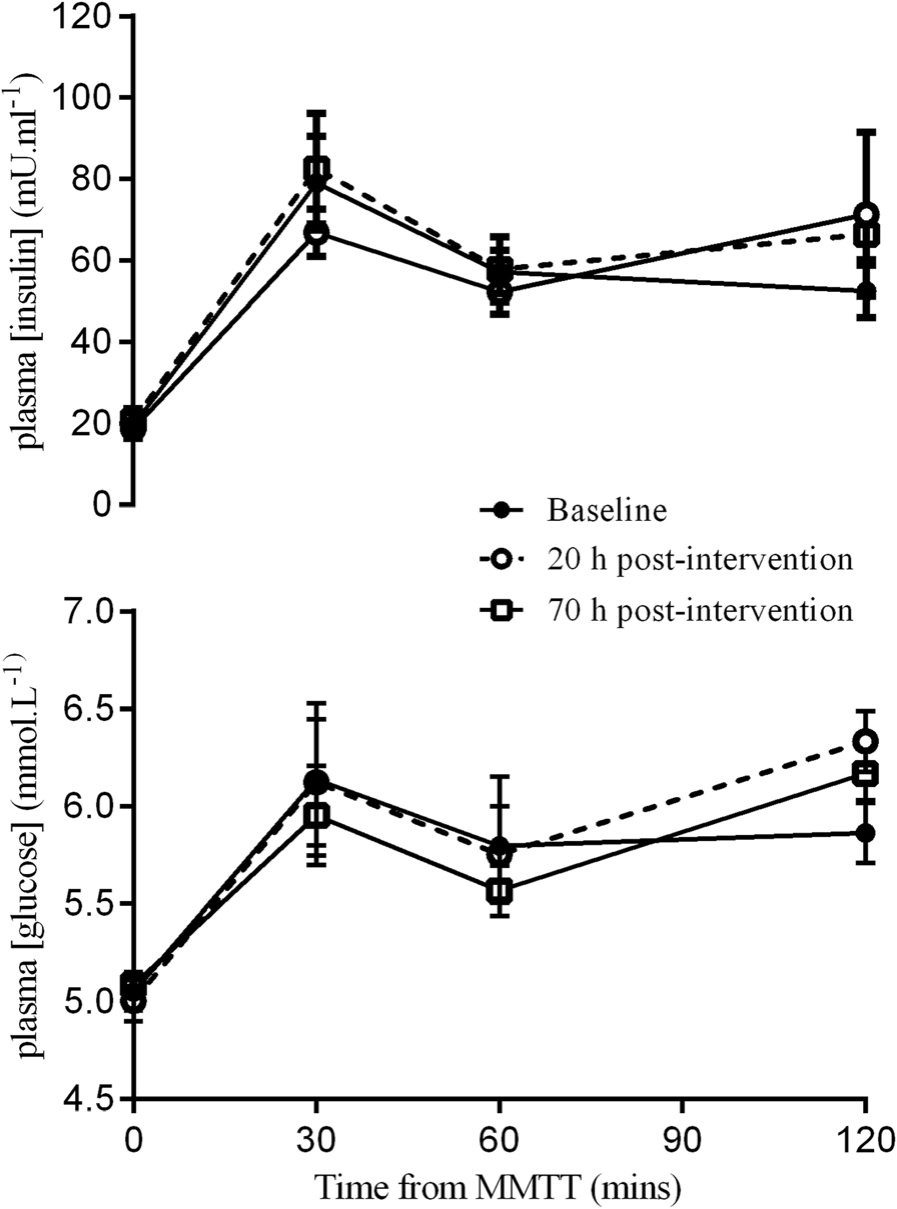
Mixed meal tolerance test: Postprandial plasma glucose and insulin response to the mixed meal tolerance test (MMTT) at baseline and at 20 h and 70 h after the HIIT intervention. Results shown as mean ± SEM

Fasting and postprandial outcomes and cardiorespiratory fitness data are shown in Table 3. There were no differences in fasting plasma glucose, insulin, QUICKI, FGIR, HOMA-IR, HOMA S% and HOMA β% at PRE, 20-POST and 70-POST intervention . The plasma glucose and insulin response during the postprandial period following the MMTT are shown in Fig. 1. There were no differences in tAUC and iAUC for glucose and insulin at PRE, 20 h and 70- POST intervention (*P >* 0.05 $\dot{V}$O_2_ max and PP output were unchanged POST compared to PRE (*P* > 0.05).

**Table 3:** Physical and biochemical characteristics at PRE, 20 h and 70 h post intervention

	PRE	20 h post intervention	70 h post intervention	*P*-value	*Effect size*
Fasting					Partial Eta Squared
Glucose (mmo∙L^−1^)	5.05 ± 0.3	5.00 ± 0.3	5.09 ± 0.2	0.86	0.028
Insulin (μU∙ml.)	19.41 ± 8.4	18.63 ± 3.5	20.60 ± 8.2	0.84	0.025
HOMA-IR (arbitrary units)	2.47 ± 1.04	2.37 ± 0.45	2.61 ± 0.99	0.85	0.027
HOMA-S% (arbitrary units)	45.86 ± 15.44	43.51 ± 8.62	42.27 ± 13.11	0.87	0.024
HOMA- B% (arbitrary units)	170.93 ± 39.86	172.70 ± 18.10	177.26 ± 46.70	0.93	0.013
QUICKI (arbitrary units)	0.311 ± 0.017	0.311 ± 0.010	0.308 ± 0.015	0.89	0.020
FGIR (mg/10^− 4^ U)	5.27 ± 1.64	4.96 ± 0.82	4.92 ± 1.43	0.86	0.025
MMTT					
iAUC Glucose (mmol∙min∙L^− 1^)	91.08 ± 80.26	107.79 ± 77.26	81.06 ± 43.99	0.57	0.089
tAUC Glucose (mmol∙min∙L^− 1^)	696.76 ± 74.11	707.67 ± 48.73	690.47 ± 36.96	0.56	0.093
iAUC Insulin (μU∙ml∙min^− 1^)	4499.57 ± 1834.26	4538.14 ± 1882.24	4908.71 ± 1329.51	0.78	0.041
tAUC Insulin (μU∙ml∙min^− 1^)	6807.00 ± 1415.10	6774.29 ± 1661.46	7380.29 ± 1906.95	0.60	0.082
Fitness					Cohen’s D
$\dot{V}$O_2_ max (L∙min^−1^)	2.44 ± 0.70	–	2.52 ± 0.76	0.27	0.10
HR max (b min^− 1^)	192 ± 8	–	193 ± 9	0.65	0.16
GET (L∙min^− 1^)	1.33 ± 0.29	–	1.35 ± 0.28	0.85	0.04
GET (% $\dot{V}$O_2_ max)	55.7 ± 7.1	–	54.9 ± 7.5	0.60	0.09
PP (W)	233 ± 58	–	244 ± 66	0.09	0.14

Results shown as mean ± SD

*HOMA-IR* homeostatic assessment of insulin resistance, *HOMA-S%* homeostatic assessment of insulin sensitivity, *HOMA- B*% %, homeostatic assessment of beta-cell function, *QUICKI* quantitative insulin sensitivity check index, *FGIR* fasting glucose;insulin ratio, *iAUC* incremental area under curve, *tAUC* total area under curve, *HR* heart rate, *GET* gas exchange threshold, *PP* peak power. *P*-values from ANOVA for fasting and MMTT outcomes, and paired sample T-test for fitness outcomes. Effect size from Partial Eta Squared for Fasting and MMTT outcomes, and Cohen’s D for fitness outcomes

**Fig. 2:**
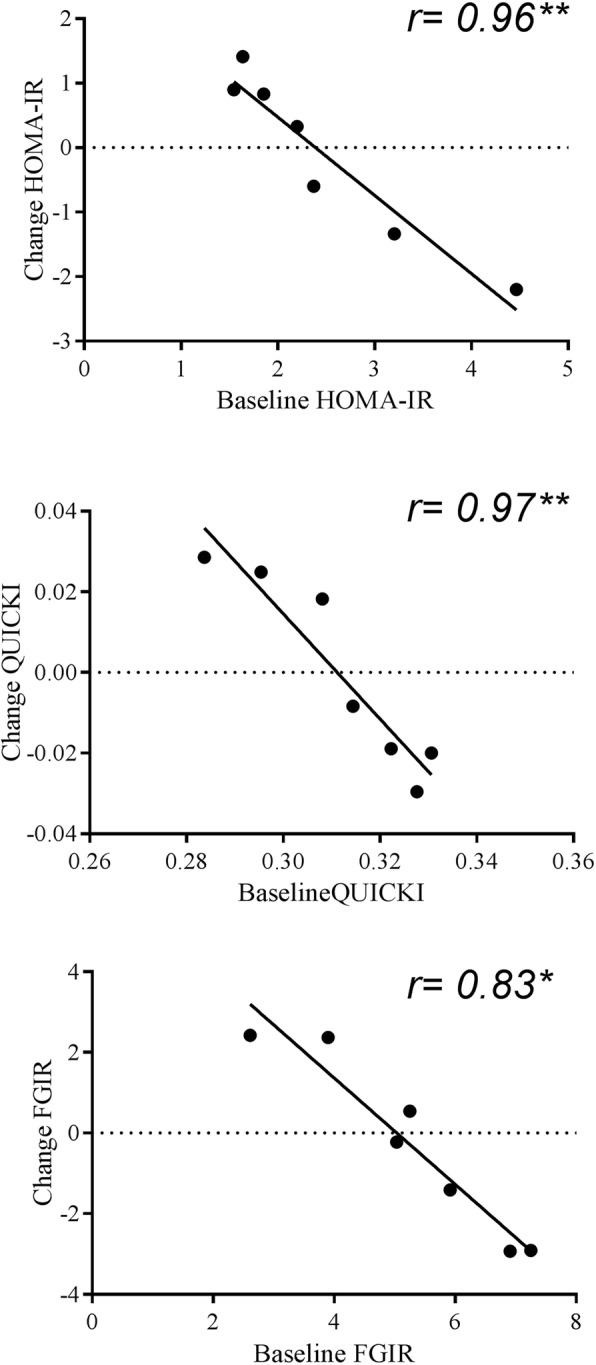
Correlations between changes in IS indices: Scatter plot showing correlation between change at 20 h POST HIIT and at baseline for Homeostatic Model Assessment of Insulin Resistance (HOMA-IR); Quantitative insulin-sensitivity check index (QUICKI) and Fasting Glucose to Insulin Ratio (FGIR). ** *P* < 0.01 *P < 0.05

Significant strong negative correlations were found between change in HOMA-IR, QUICKI and FGIR 20-POST and PRE HOMA-IR, QUICKI and FGIR (*r =* − 0.96, *P* = 0.001; *r =* − 0.97, *P* = 0.001; *r =* − 0.83, *P* = 0.022 for HOMA-IR, QUICKI and FGIR respectively, Fig. 2). The changes in HOMA-IR, QUICK and FGIR post intervention were not related to $\dot{V}$O_2_ max or BMI (both *P >* 0.05). There was no correlation between changes in postprandial outcomes at 20-POST and PRE training values (*P* > 0.05 for all).

## Discussion

The key finding of this preliminary pilot study was that 2 weeks of HIIT did not elicit any acute or chronic changes to fasting and postprandial markers of metabolic health in a group of adolescent boys. However, a strong negative correlation was found between baseline IR (HOMA-IR, QUICKI and FGIR) and the change at 20-POST HIIT, suggesting a beneficial effect in participants with the greatest IR at baseline. Short duration HIIT protocols may therefore be a useful exercise strategy for youth with poorer metabolic health profile at baseline.

In the present study 2 weeks of HIIT (8–10 1 min intervals at ~ 90% of PP, interspersed with 75 s of unloaded pedalling) was not sufficient to improve IR or fasting and postprandial measures of metabolic health when measured 20 h or 70 h after the final training session. Interestingly, our findings corroborate those of earlier studies conducted on healthy, asymptomatic adolescents. In two separate studies [42, 43], Buchan and colleagues reported no change to either fasting insulin or glucose after a 7 week school-based HIIT program (4–6 repeats of 30 s maximal sprints with 20–30 s recovery 3 x per week), but did not report HOMA index of IR, QUICKI or FGIR. However, in these studies moderate intensity PA did improve fasting insulin suggesting this intensity of exercise may be superior to HIIT. Similarly, an exploratory study of a 10 week school based HIIT programme for healthy adolescent showed no changes to glucose [44]. In a 5 week HIIT intervention consisting of 10 × 1 min sprints 3 x /week Van Bijon and colleagues [45] reported a trend towards improvement for fasting glucose, but not insulin in healthy 10–13 year olds.

In contrast, studies investigating the effectiveness of HIIT in overweight or obese participants over 12 weeks [13–15, 17] have shown improvements to fasting glucose, insulin and HOMA-IR. These findings may show that the duration of the HIIT programme is important as HIIT programmes lasting > 12 weeks have yet to be conducted in normal weight adolescents to our knowledge. However, it is pertinent to note that in these HIIT studies in overweight and obese youths (15, 27, 36) the participants had a baseline HOMA-IR of ~ 4–5 arbitrary units (AU), which is notably higher than the present study (2.5 ± 1.0 AU) and suggests a limited window to improve IR after HIIT in participants with low baseline IR. Published reference values for HOMA-IR in Caucasian youth suggest a 75th percentile cut-off point for cardiometabolic risk at 3.02 AU [46]. In our study, analysis of the individual data found three participants appeared to respond positively to 2 weeks of HIIT and were characterized by an IR between the 90th and 97th centile. These participants recorded an improvement in IR 20- POST ranging from 59 to 219%, with the largest improvement occurring in the participant with the highest baseline HOMA-IR. This is reflected by the significant negative correlation between the change in IR 20- POST and PRE IR (Fig. 2) which was evident in HOMA-IR, QUICKI and FGIR and suggests that 2 weeks of HIIT may be a feasible intervention to improve metabolic health in adolescents with a high IR at baseline. Finally, it has recently been reported that the ability for physical activity to attenuate IR is diminished in 16 year-old adolescents [10]. The mean age of participants in the present study was 14.3 y with pubic hair stages between 3 and 4, which may have influenced the effectiveness of the HIIT intervention to modify plasma glucose and insulin. Taken collectively, there may be a limit to improvements to IR through just 2 weeks of HIIT, especially in those who have a low IR at baseline, are or normal weight and in late adolescence.

In the current study, 2 weeks of HIIT had no effect on postprandial plasma glucose and insulin after a MMTT. The inclusion of postprandial measures is a strength of our study because it is known that postprandial hyperglycaemia is a contributor to glycaemic control (e.g. HbA_1c_), which often precedes any increase in fasting glucose levels and is more harmful to skeletal muscle glucose homeostasis than chronically sustained hyperglycaemia [47]. In overweight/obese adolescents reductions in 2 h postprandial glucose and insulin after an oral glucose tolerance test (OGTT) have been shown after 12 weeks of HIIT, but not after matched-duration moderate intensity exercise training [15]. In healthy young men (21 ± 2 y), Babraj and colleagues [48] found 2 weeks of HIIT (6 sessions of 4–6 30 s sprints) reduced the plasma glucose and insulin AUC response to an OGTT by 12 and 37% respectively, 2 to 3 days after the last exercise session. In agreement with the present study, however, the authors found no changes to fasting glucose or insulin [48]. These findings suggest that the response to exercise training may differ for fasting and dynamic (postprandial) measures of insulin and glucose, which we have also found in previous work [21]. Thus it is possible that the use of the MMTT to examine postprandial changes in glucose and insulin rather than an OGTT in the current study may account for the lack of effect when compared to the work by Babraj and colleagues. In particular, the MMTT will have a lower glycaemic index which will alter the glucose excursions [49] and is likely to have influenced the rate of glucose appearance in the circulation [50]. That said the MMTT holds better external validity as it is more representative of the habitual nutrient meal composition compared to an OGTT.

One of the aims of this study was to highlight any acute benefits from the HIIT by measuring the outcomes 20 h post the final training session. Contrary to our original hypothesis, no acute improvements in fasting or postprandial glucose and insulin were present at ~ 20-POST. We have previously shown that a single bout of HIIE can improve both glucose tolerance and IS in adolescent boys [18], and that these changes persist for up to 24 h after exercise [21]. It is therefore surprising that 2 weeks of HIIT did not improve metabolic outcomes the day after the last training session in the current study. However, the aforementioned acute exercise studies used an OGTT and not a MMTT, which may account for the discrepancies in findings. The lack of change to metabolic outcomes 20-POST in the current study may also indicate that improvements after HIIT in healthy adolescents do not persist into the next day.

Aerobic fitness, as measured using a validated cycle test to exhaustion, was unchanged in adolescent boys after the 2 week HIIT programme. This result differs from the outcome of a recent meta-analysis showing that ≥4 weeks of HIIT to have a large effect on improving aerobic fitness (ES = 1.05) in adolescents [51]. A 5% improvement in $\dot{V}$O_2_ max has been shown after 2 weeks of HIIT, however this study incorporated 30 s “all out” sprint type HIIT [12], which may have provided a greater stimulus to augment $\dot{V}$O_2_ max.

This study is the first to asses both fasting and postprandial measures of metabolic health in a healthy adolescent population after short duration HIIT programme. Previous studies in this area are largely limited to overweight/obese adolescents and longer duration HIIT programmes. The strengths of this study include the control of physical activity and diet prior to the experimental measures, which limits any confounding effects of these factors. Additionally we include multiple indices on IR, which in previous work is limited to HOMA-IR, this is important as we have recently shown HOMA-IR to have a large variability in this population, with other measures such as QUICKI and FGIR potentially better placed to use in this population [26] . Limitations include the lack of a control group, although this is consistent with other short duration HIIT studies in youth [12] and adults [52]. The small sample size is also a limitation; however this study is reported as a pilot study. Future work should investigate the potential of HIIT interventions targeted at adolescents with impaired insulin resistance (rather than weight status) with a larger sample size. Based on the observed effect size in this study, and previous reliability work [26] we would estimate a sample of ~ 75 boys to see changes HOMA-IR.

## Conclusion

This preliminary study shows that fasting or postprandial measures of insulin and glucose in adolescents were not sensitive to change after 2 weeks of HIIT. However, a strong negative correlation between baseline IR and change in IR after HIIT, but not for BMI suggests the potential for this type of intervention to promote metabolic health in a individuals with elevated baseline IR, who are at risk of developing type two diabetes.

## Data Availability

The datasets generated and analysed during the current study are not publicly available due to ethical restrictions but are available from the corresponding author upon reasonable request.

## Abbreviations

CVD: Cardiovascular disease
FGIR: Fasting glucose:insulin ratio
GET: Gas exchange threshold
GLUT-4: Skeletal muscle glucose transporter 4
HIIE: High-intensity interval exercise
HIIT: High-intensity interval training
HOMA-IR: Homeostatic model assessment of insulin resistance
iAUC: Incremental area under the curve
IR: Insulin resistance
IS: Insulin sensitivity
MMTT: Mixed Meal Tolerance Test
MVPA: Moderate to vigorous physical activity
$\dot{V}$O_2_ max: Maximal oxygen uptake
PA: Physical activity
PP: Peak power
QUICKI: Quantitative insulin sensitivity check index
T2D: Type two diabetes
tAUC: Total area under the curve
